# Clinical Characteristics Associated with Return Visits to the Emergency Department after COVID-19 Diagnosis

**DOI:** 10.5811/westjem.2021.9.52824

**Published:** 2021-11-05

**Authors:** Iltifat Husain, James O’Neill, Rachel Mudge, Alicia Bishop, K. Alexander Soltany, Jesse Heinen, Chase Countryman, Dillon Casey, David Cline

**Affiliations:** Wake Forest School of Medicine, Department of Emergency Medicine, Winston-Salem, North Carolina

## Abstract

**Introduction:**

Patients diagnosed with coronavirus disease 2019 (COVID-19) require significant healthcare resources. While published research has shown clinical characteristics associated with severe illness from COVID-19, there is limited data focused on the emergency department (ED) discharge population.

**Methods:**

We performed a retrospective chart review of all ED-discharged patients from Wake Forest Baptist Health and Wake Forest Baptist Health Davie Medical Center between April 25–August 9, 2020, who tested positive for severe acute respiratory syndrome-coronavirus-2 (SARS-CoV-2) from a nasopharyngeal swab using real-time reverse transcription polymerase chain reaction (rRT-PCR) tests. We compared the clinical characteristics of patients who were discharged and had return visits within 30 days to those patients who did not return to the ED within 30 days.

**Results:**

Our study included 235 adult patients who had an ED-performed SARS-CoV-2 rRT-PCR positive test and were subsequently discharged on their first ED visit. Of these patients, 57 (24.3%) had return visits to the ED within 30 days for symptoms related to COVID-19. Of these 57 patients, on return ED visits 27 were admitted to the hospital and 30 were not admitted. Of the 235 adult patients who were discharged, 11.5% (27) eventually required admission for COVID-19-related symptoms. With 24.3% patients having a return ED visit after a positive SARS-CoV-2 test and 11.5% requiring eventual admission, it is important to understand clinical characteristics associated with return ED visits. We performed multivariate logistic regression analysis of the clinical characteristics with independent association resulting in a return ED visit, which demonstrated the following: diabetes (odds ratio [OR] 2.990, 95% confidence interval [CI, 1.21–7.40, P = 0.0179); transaminitis (OR 8.973, 95% CI, 2.65–30.33, P = 0.004); increased pulse at triage (OR 1.04, 95% CI, 1.02–1.07, P = 0.0002); and myalgia (OR 4.43, 95% CI, 2.03–9.66, P = 0.0002).

**Conclusion:**

As EDs across the country continue to treat COVID-19 patients, it is important to understand the clinical factors associated with ED return visits related to SARS-CoV-2 infection. We identified key clinical characteristics associated with return ED visits for patients initially diagnosed with SARS-CoV-2 infection: diabetes mellitus; increased pulse at triage; transaminitis; and complaint of myalgias.

## INTRODUCTION

In December 2019 a pathologic human coronavirus, severe acute respiratory syndrome coronavirus 2 (SARS-CoV-2), emerged in Wuhan, China, causing coronavirus disease 2019 (COVID-19). In less than a year since its emergence, more than 730,368 deaths have been attributed to COVID-19 in the United States (US) with over 45,149,234 total cases reported.^1^ Patients diagnosed with COVID-19 present not only a diagnostic challenge for the emergency department (ED), but also require significant healthcare resources.^2^ One of the diagnostic challenges emergency physicians face is the prolonged clinical course of COVID-19. The median time from onset of illness to acute respiratory distress syndrome is 8–12 days, with the median time of onset of illness to intensive care unit admission 9.5–12 days. This variability in clinical course makes it difficult for emergency physicians to predict whether patients diagnosed with COVID-19 in the ED will have a return visit or admission. While published research has shown clinical characteristics associated with severe illness from COVID-19, there is limited data focusing on the ED discharge population.^3^,^4^

Significant hospital resources and operational changes are required to manage patients who present to the ED with symptoms concerning for COVID-19. These include use of personal protective equipment (PPE), negative pressure rooms, cohorting of patients, and more.^5^ In October–November 2020, a significant increase in COVID-19 was experienced in the ED setting. The US Centers for Disease Control and Prevention reported coronavirus-like illness (CLI) or a COVID-19 diagnostic code in the ED setting increasing from 2.7% of visits in early October to as high as 6.6% in late November 2020. In some states, such as New Mexico, CLI or COVID-19 diagnostic code visits have been as high as 16.5% of ED visits.^6^ This dramatic increase in COVID-19 diagnoses makes it critical to understand the clinical characteristics of these patients and how many may have return ED visits.

Currently there are no published reports of the clinical characteristics of patients who are discharged from the ED with a SARS-CoV-2-positive test and return within 30 days. Understanding these clinical characteristics would allow EDs to better prepare for return visits and allocate resources to help these patients in the outpatient setting once they are discharged. With EDs and hospitals experiencing constrained capacity, these proactive measures could enable hospital systems to reduce return visits of patients with COVID-19 and improve operational planning for them.

## METHODS

We conducted a retrospective chart review of all ED-discharged patients from the Wake Forest Baptist Health and Wake Forest Baptist Health Davie Medical Center who had an ED-positive laboratory SARS-CoV-2 real-time reverse transcription polymerase chain reaction (rRT-PCR) test resulting from a nasopharyngeal swab between April 25–August 9, 2020. We compared the clinical characteristics of patients who were discharged and had return visits within 30 days to those patients who did not return to the ED within 30 days. This study was approved by the Biomedical Institutional Review Board of Wake Forest School of Medicine.

Of patients discharged from the ED with positive rRT-PCR testing, we included patients aged 18 and older. Patients’ health records underwent individual chart review to determine whether they had a return visit to the ED within 30 days for COVID-19-related symptoms. Patients who did not have a return visit to the ED within 30 days for COVID-19 related symptoms comprised our control cohort of no return ED visits. We analyzed the data using SAS 9.4 (SAS Institute, Inc., Cary, NC). Chi-square test was used to compare frequencies of categorical variables between discharged ED patients with a positive ED rRT-PCR for SARS-CoV-2 who returned after their index ED visit and those patients who did not return. We used Student’s t-tests or Wilcoxon signed-rank tests to compare continuous variables between groups. Logistic regression was used for multivariate analysis of those variables that were independently associated with return to the ED.

## RESULTS

Our study included 235 adult patients who had an ED-performed SARS-CoV-2 rRT-PCR positive test and were subsequently discharged on their first ED visit. Of these patients, 57 (24.3%) had return visits to the ED within 30 days for symptoms related to COVID-19. Of these 57 patients, on return ED visits 27 were admitted to the hospital and 30 were not admitted. Of the 235 adult patients who were discharged, 11.5% (27) eventually required admission for COVID-19 related symptoms. With 24.3% of patients having a return ED visit after a positive SARS-CoV-2 test and 11.5% requiring eventual admission, it is important to understand clinical characteristics associated with return ED visits.

Table 1 lists clinical characteristics and their univariate association with return to the ED. The chronic conditions that we found significantly associated with return ED visits were diabetes (OR 3.06, 95% CI, 1.52–6.13, *P* = 0.002) and hypertension (OR 2.18, 95% CI, 1.17–4.05, *P* = 0.013). Patients between ages 50–69 were more likely to have a return ED visit (OR 1.89, 95% CI, 1.02–3.50, *P* = 0.042). While patients with return ED visits had a higher percentage of abnormal chest radiographs at their index ED visit than those who did not return (42.1% to 28.1%), this was not statistically significant. Lab abnormalities significantly associated with higher return visits were transaminitis (OR 3.99, 95% CI, 1.53–10.4, *P* < 0.001); thrombocytopenia (OR 3.0, 95% CI, 1.2–7.2, *P* = 0.012); and abnormal glomerular filtration rate (OR 4.1, 95% CI, 1.2–13.9, *P* = 0.025). Interestingly, diagnostic markers used for risk stratification, such as D-dimer and lymphopenia, were not significantly associated with higher return visits to the ED. Neither were health insurance status or race significantly associated with higher return visits to the ED. We analyzed triage and discharge vital signs from patient index visits and found heart rate ≥ 90 during triage and discharge was significantly associated with return ED visits.

Table 2 lists those clinical characteristics that retained independent association with a return visit to the ED after the index visit to the ED due to COVID-19. These clinical characteristics included increased pulse at triage, (OR 1.043, 95% CI, 1.020–1.065, *P* = 0.0002; myalgia, (OR 4.427, 95% CI, 2.028–9.663, *P* = 0.0002; history of diabetes mellitus, (OR 2.990, 95% CI, 1.208–7.403, *P* = 0.0179; and transaminitis (OR 8.973, 95% CI, 2.654–30.333, *P* = 0.0004. Transaminitis was defined as any abnormal elevation in aspartate aminotransferase or alanine aminotransferase above the laboratory-defined upper limit of normal.

## DISCUSSION

Our study shows the key clinical characteristics associated with ED return visits for patients discharged with ED-positive SARS-CoV-2 testing. After controlling for other clinical characteristics, multivariate logistic regression found that history of diabetes mellitus, a complaint of myalgia, an increased pulse at triage, and transaminitis were independently associated with a return ED visit. As EDs across the country continue to treat COVID-19 patients, it is important to understand clinical factors associated with return visits to prevent unnecessary COVID-19 return visits. The clinical characteristics we found associated with ED return visits will need to be validated independently. This analysis is part of a forthcoming study encompassing multiple EDs and a larger patient population.

We encourage hospital operational teams to focus on the ED-discharge patient populations we have identified in our study to proactively prepare and attempt to prevent unnecessary ED evaluations in a time when hospital capacity is limited.

## LIMITATIONS

Limitations of our study include possible sample bias. Our study population was made up of 49.3% Hispanics, 21.6% Black/non-Hispanic, and 26.4% White/non-Hispanic. However, this high percentage of Hispanics and Black/non-Hispanic does correlate with known disproportionate rates of SARS-CoV-2 infection in the US by race.^7^ A second limitation is the study’s duration (106 days) and the number of total patients (235).

## CONCLUSION

Our study identified key clinical characteristics associated with return ED visits for patients initially diagnosed with SARS-CoV-2 infection: diabetes mellitus; increased pulse at triage; transaminitis; and complaint of myalgias.

## Figures and Tables

**Table 1:** Univariate analysis: clinical characteristics of patients with return visits to the emergency department after being diagnosed with coronavirus 2019.

Clinical characteristics (Initial ED visit)	Total N = 235	No return ED visit N = 178 N (% cohort)	Return ED visit N = 57 N (% cohort)	Odds ratio (95% CI)	Standard error	*P*-value
Age						
18–49	151	120 (67.4)	31(54.4)	0.58 (0.31–1.06)	0.31	0.076
50–69	77	52 (29.2)	25 (43.9)	1.89 (1.02–3.50)	0.31	0.042
>70	7	6 (3.3)	1 (1.8)	1.16 (0.39–3.28)	0.54	0.827
Chronic conditions						
Asthma or COPD	30	24 (13.5)	6 (10.5)	0.76 (0.29–1.95)	0.48	0.561
Diabetes	44	25 (14.0)	19 (33.3)	3.06 (1.52–6.13)	0.35	0.002
Hypertension	72	47 (26.4)	25 (43.9)	2.18 (1.17–4.05)	0.32	0.013
Vital signs						
HR < 89 (triage)	96	80 (44.9)	16 (28.1)	0.478 (0.24–0.91)	0.33	0.026
HR > 90 (triage)	139	98 (55.1)	41 (71.9)	2.09 (1.09–4.00)	0.33	0.026
HR < 89 (discharge)	168	138 (77.5)	30 (52.6)	0.322 (0.17–0.60)	0.32	0.001
HR > 90 (discharge)	67	40 (22.5)	27 (47.4)	3.11 (1.66–5.82)	0.32	0.001
Lab/imaging						
Chest CXR normal	88	69 (38.8)	19 (33.3)	0.79 (0.41–1.47)	0.32	0.461
Chest CXR abnormal	74	50 (28.1)	24 (42.1)	1.86 (1.00–3.45)	0.32	0.047
Chest CXR not ordered	73	59 (33.1)	14 (24.6)	0.65 (0.33–1.29)	0.34	0.225
Transaminitis	19	9 (5.1)	10 (52.6)	3.99 (1.53–10.4	0.48	<0.0001
Thrombocytopenia	24	13 (7.3)	11 (19.3)	3.0 (1.2–7.2)	0.44	0.012
Lymphopenia	34	24 (13.5)	10 (17.5)	1.36 (0.6–3.6)	0.41	0.449
D-dimer positive	9	7 (3.9)	2 (3.5)	0.88 (0.17–4.4)	0.81	0.885
GFR abnormal	11	5 (2.8)	6 (10.5)	4.1 (1.2–13.9)	0.62	0.025
Labs not ordered	109	89 (50.0)	20 (35.1)	0.541 (0.29–1.00)	0.31	0.051
Health insurance (Y)	112	81 (45.5)	31 (54.4)	1.47 (0.80–2.67)	0.31	0.212
Weight						
BMI < 25	59	49 (27.5)	10 (17.5)	0.56 (0.26–1.19)	0.39	0.134
Overweight (BMI 25 to 29.9)	52	38 (21.3)	14 (24.6)	1.2 (0.60–2.42)	0.36	0.611
Obesity (BMI > 30)	124	91 (51.1)	33 (57.9)	1.31 (0.72–2.40)	0.31	0.375
Race/Ethnicity						
Asian, non-Hispanic	5	4 (2.2)	1 (1.8)	0.777 (0.08–7.10)	0.17	0.746
Black, non-Hispanic	50	37 (20.8)	13 (22.8)	1.13 (0.55–2.31)	0.37	0.746
Other race, non-Hispanic	5	3 (1.7)	2 (3.5)	2.21 (0.35–13.02)	0.93	0.417
White, non-Hispanic	61	46 (25.8)	15 (26.3)	1.03 (0.52–2.02)	0.35	0.943
Hispanic	114	88 (49.4)	26 (45.6)	0.858 (0.47–1.56)	0.31	0.615

*ED*, emergency department; *CI*, confidence interval; *COPD*, chronic obstructive pulmonary disease; *HR*, heart rate; *CXR*, chest radiograph; *GFR*, glomerular filtration rate; *BMI*, body mass index.

**Table 2:** Multivariate logistic regression: clinical characteristics independently associated with return to the emergency department after the initial visit for COVID-19 infection.

Clinical characteristic	Odds ratio	95% Confidence interval		*P*-value
Pulse at triage (increasing)*	1.04	1.02	1.07	0.0002
Myalgia	4.43	2.03	9.66	0.0002
History of diabetes mellitus	2.99	1.21	7.40	0.0179
Transaminitis	8.97	2.65	30.33	0.0004

* Continuous variable; as pulse increased, the odds ratio increased 1.043 per each beat per minute.
